# Serum levels of IL-6 and CRP can predict the efficacy of mFOLFIRINOX in patients with advanced pancreatic cancer

**DOI:** 10.3389/fonc.2022.964115

**Published:** 2022-07-29

**Authors:** Feifei Shen, Chuan Liu, Weiguo Zhang, Sijia He, Fan Wang, Jingjue Wang, Qi Li, Fei Zhou

**Affiliations:** ^1^ Department of Oncology, Shanghai General Hospital, Shanghai Jiaotong University School of Medicine, Shanghai, China; ^2^ Department of Radiology, Shanghai General Hospital, Shanghai Jiaotong University School of Medicine, Shanghai, China

**Keywords:** C-reactive protein, interleukin-6, inflammatory markers, metastatic pancreatic cancer, modified FOLFIRINOX

## Abstract

**Objectives:**

There is an urgent need for biomarkers that predict the survival outcome of patients diagnosed with metastatic pancreatic cancer, undergoing systemic chemotherapy. This study aimed to identify biomarkers associated with the survival of mPC patients treated with modified FOLFIRINOX (mFOLFIRINOX) as first-line chemotherapy.

**Methods:**

This was a retrospective study of 30 patients with mPC who received mFOLFIRINOX between October 2018 and March 2021. Data on carcinoembryonic antigen (CEA), cancer antigen (CA)199, interleukin (IL)-6, C-reactive protein (CRP), neutrophils, platelets, lymphocytes, and albumin were collected and dichotomized using the upper or lower limit, as appropriate. These markers were examined for their association with progression-free survival (PFS). A receiver operating characteristic (ROC) curve analysis was used to explore a suitable model to predict mFOLFIRINOX effectiveness.

**Results:**

IL-6 and CRP levels were associated with poor progression (P = 0.004 and P = <0.001, respectively) of mPC. The high IL-6 level was an independent poor prognostic factor for PFS (HR=4.66, 95%CI: 1.32-16.37, P=0.016) in the multivariable analysis. Patients with high IL-6 levels had a shorter PFS than those with low IL-6 levels (median PFS: 257 vs. 150 days, P=0.020). An increase in IL-6 and CRP levels during chemotherapy positively correlated with disease progression (P = <0.001 for both). The model combining IL-6 with CRP levels helped predict the outcomes of mPC patients treated with mFOLFIRINOX (AUC: 0.811, 95%CI: 0.639-0.983, P=0.003).

**Conclusions:**

The serum levels of IL-6 and CRP might be considered as valuable biomarkers in predicting the outcomes of patients with mPC who received the mFOLFIRINOX regimen.

## Introduction

Pancreatic cancer (PC) is a very lethal malignancy, with an incidence rate nearly equal to its mortality rate (1). The situation is even worse in China, where 83,600 new PC cases were reported in 2017, and an estimated 85,100 patients died (2). This was because most patients were not diagnosed timely, and 80%–90% of them were in an advanced stage at diagnosis and were unable to receive curative resection (3). The reported 5-year survival rate of PC is only 8% (1).

Systemic chemotherapy is the standard of care for patients with metastatic PC (mPC). The FOLFIRINOX regimen [oxaliplatin, irinotecan, leucovorin, and 5-fluorouracil (5-FU)] significantly improves overall survival (OS) compared with gemcitabine alone in patients with mPC, but it is associated with higher toxicity (4). The modified FOFIRINOX (mFOLFIRINOX) regimen was, therefore, suggested with the intention of reducing toxicity while preserving efficacy (5–7). It was also proven to be well tolerated and effective in Chinese patients (7). The overall response rate (ORR) of mFOLFIRINOX was reported to be 30%–53.5% (5–7). Although the TNM stage and serum carbohydrate antigen 199 (CA199) levels have been demonstrated to be associated with survival, their value in predicting the efficacy of mFOLFIRINOX is low (8, 9). Therefore, biomarkers that can effectively identify patients with mPC who will benefit from mFOLFIRINOX are urgently needed to improve patient management.

Inflammation predisposes individuals to the initiation, growth, progression, and metastatic spread of cancer (10). Inflammatory markers are associated with the prognosis of PC (11–19). One study showed that elevated serum C-reactive protein (CRP) levels were significantly associated with poor clinical outcomes in patients with PC (11), while another study found that the CRP-to-albumin ratio could be a significant and promising inflammatory prognostic score (12). In addition, many studies suggested that the changes in interleukin (IL) expression were associated with poor prognosis in advanced PC (APC) (13, 14). Furthermore, high CRP and IL-6 levels were associated with a poor response in patients with PC receiving first-line gemcitabine monotherapy (15, 16). Higher levels of tumor-infiltrating neutrophils were significantly associated with shorter survival, whereas higher levels of tumor-infiltrating lymphocytes, as well as low levels of platelets (PLT), were described as being positively correlated with extended OS and progression-free survival (PFS) in PC (17–19).

Therefore, it was hypothesized that inflammatory markers, such as CRP, ILs, tumor-infiltrating neutrophils and lymphocytes, and platelets, might be potential indicators for predicting the response of patients with mPC to mFOLFIRINOX. This study aimed to investigate the impact of these inflammatory markers on the outcomes of first-line mFOLFIRINOX chemotherapy in patients with mPC. The results of this study could help improve the management of mPC by identifying the patients who might benefit the most from mFOLFIRINOX.

## Materials and methods

### Study design and patients

**Figure 1:**
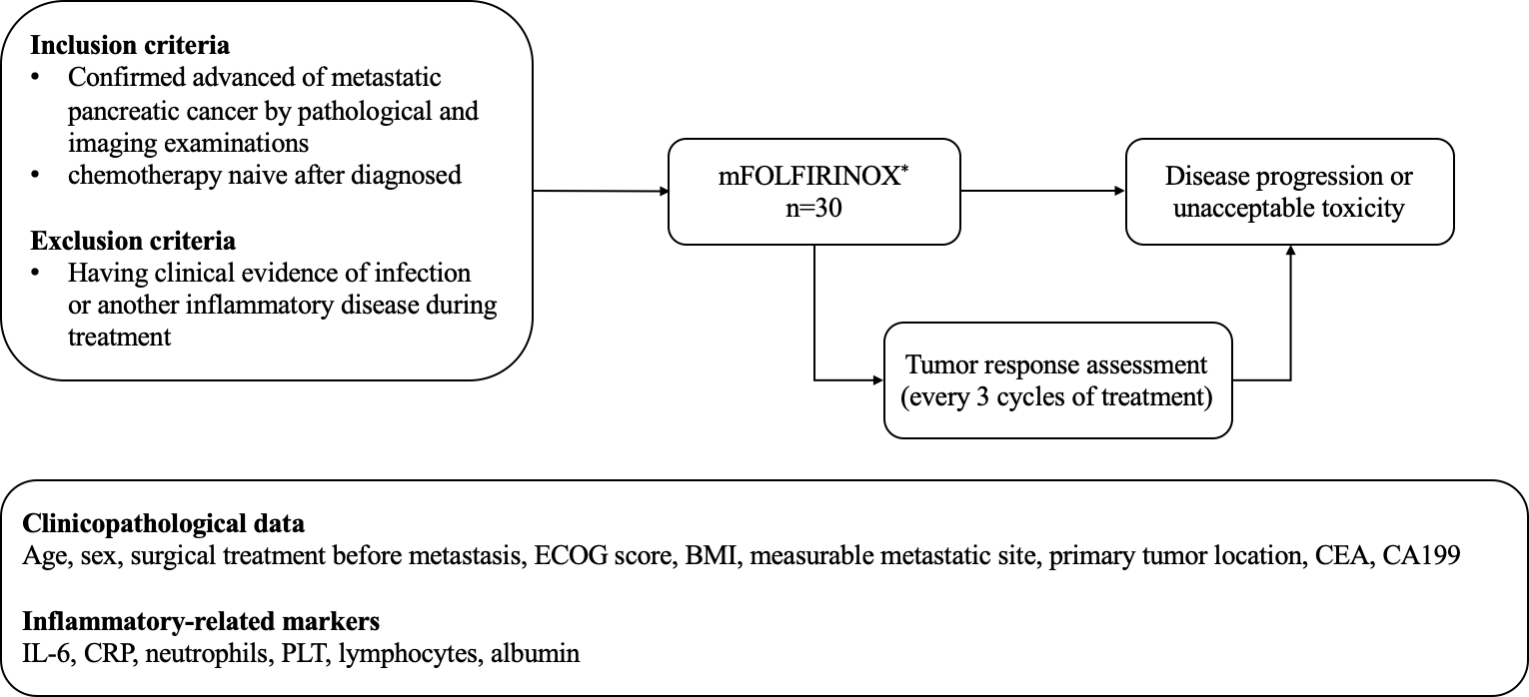
The mFOLFIRINOX regimen: oxaliplatin 68 mg/m^2^ given as a 2-h intravenous infusion, then leycovorin 400 mg/m^2^ delivered as a 2-h intravenous infusion, qnd finally 5-FU 2400 mg/m^2^ given as a continous intravenous infusion over a 46-h period. The regimen had beeb administered on day 1 and the repeatedevery 2 weeks until disease progression or patient refusal. ECOG, Easteran Cooperative Oncology Group; BMI, body mass indez; CEA, carcinoembryonic antigen; CA199, cancerantigen199; IL-6 interleukin-6; CRP, C-reactive protein; PLT, platelets.

This was a retrospective observational single-center study that included patients treated with mFOLFIRINOX at Shanghai General Hospital between October 2018 and March 2021. All patients included had been diagnosed with mPC by pathological and imaging examinations and had received mFOLFIRINOX as first-line chemotherapy. The patients who had clinical evidence of infection or another inflammatory disease during treatment were excluded (**Figure 1**). The study was approved by the ethics board of Shanghai General Hospital. The requirement for informed consent was waived by the committee due to the retrospective nature of the study.

### Systemic chemotherapy

The mFOLFIRINOX regimen (oxaliplatin 68 mg/m^2^ given as a 2-h intravenous infusion, immediately followed by irinotecan 135 mg/m^2^ given as a 90-min intravenous infusion, then leucovorin 400 mg/m^2^ delivered as a 2-h intravenous infusion, and finally 5-FU 2400 mg/m^2^ given as a continuous intravenous infusion over a 46-h period) had been administered on day 1 and then repeated every 2 weeks until disease progression or patient refusal. Dose modification was allowed when unacceptable toxicity occurred.

### Response assessment

Tumor response assessment was examined every three cycles of treatment by computed tomography (CT) or magnetic resonance imaging (MRI). The efficacy outcomes included complete response (CR), partial response (PR), progressive disease (PD), and stable disease (SD) according to the Response Evaluation Criteria in Solid Tumors version 1.1 (RECIST 1.1) (20). The best clinical response during chemotherapy for each patient was recorded as the tumor response. ORR was calculated as CR + PR/all evaluated patients. The disease control rate (DCR) was calculated as CR + PR + SD/all evaluated patients.

### Data collection

The clinicopathological data including age, sex, surgical treatment before metastasis (yes or no), Eastern Cooperative Oncology Group (ECOG) score, body mass index (BMI), measurable metastatic site, primary tumor location, carcinoembryonic antigen (CEA), and CA199 were collected from the patients’ records. The other inflammatory-related markers, including IL-6, CRP, neutrophils, PLT, lymphocytes, and albumin, were also collected from the records. Blood samples had been obtained within 3 days before initiating treatment or during chemotherapy. CRP had been measured with an immunoturbidimetry assay. Serum IL-6, CA199, and CEA levels had been determined using electro-chemiluminescence immunoassay. As shown in **Table 1**, the normal reference value of IL-6 and CRP was 0–7 pg/mL and 0–10 mg/L, respectively. The other reference values were 0–40 U/mL for CA199, 0–5 ng/mL for CEA, 35–50 g/L for albumin, 85−303 × 10^9^ for PLT, 2–7 × 10^9^ for neutrophils, and 0.8–4 × 10^9^ for lymphocytes. Using the upper limits of normal for IL-6 (>7 pg/mL), CRP (>10 mg/L), PLT (>303 × 10^9^/L), neutrophils (>7 × 10^9^/L), CA199 (>40 U/mL), and CEA (>5 ng/mL) as cutoff values, the patients were classified with low or high values for each of these markers. Using the lower limit of normal for lymphocytes (<0.8×10^9^/L) and albumin (<35 g/L) as cutoff values, the patients were classified with low and high values. The definitions and cutoff values were based on previous reports (21–23). CEA, CA199, CRP, IL-6, neutrophils, PLT, lymphocytes, and albumin were tested at three-cycle intervals, at the same time as response evaluation. A dynamic increase was defined as two or more consecutive increases in the levels of tested markers compared with the last testing without a cutoff threshold. All markers described earlier were measured by the Department of Laboratory Medicine of Shanghai General Hospital (Shanghai Jiaotong University School of Medicine, Shanghai, China (using standard routine laboratory methods).

**Table 1:** The normal reference and cutoff values of serum markers.

	normal reference value	High	Low
IL-6 (pg/mL)	0 – 7	> 7	≤ 7
CRP (mg/L)	0 – 10	> 10	≤ 10
PLT (×10^9^)	85 − 303	> 303	≤ 303
Neutrophil (×10^9^)	2 – 7	> 7	≤ 7
CA199 (U/mL)	0 – 40	> 40	≤ 40
CEA (ng/mL)	0 – 5	> 5	≤ 5
Lymphocytes (×10^9^)	0.8 – 4	≥ 0.8	< 0.8
albumin (g/L)	35 – 50	≥ 35	< 35

IL-6, interleukin-6; CRP, C-reactive protein; PLT: Platelets; CEA, carcinoembryonic antigen; CA199, Carbohydrate antigen 199.

Survival data were obtained from the medical charts. PFS was defined as the time from the start of chemotherapy to documented disease progression or death, whichever occurred first.

### Statistical analysis

Statistical analysis was performed using SPSS 20.0 (IBM Corp., NY, USA). The relationships between inflammatory markers and the clinical response were assessed using Fisher’s exact test (for categorical variables) and Student’s *t* test (for continuous variables). The correlations between dynamically changing inflammatory markers and the clinical response were evaluated using Spearman’s rank correlation coefficient. Survival analysis was performed using the Kaplan-Meier method, and the curves were compared using the log-rank test. Multivariate analysis was performed using a stepwise forward Cox regression (likelihood ratio, enter *P* < 0.05, remove *P* > 0.10) with significant markers from the univariate analyses (*P* < 0.05); the results were presented as hazard ratio (HR) and 95% confidence interval (CI). Receiving operating characteristic (ROC) curve analysis was used to explore a combination of biomarkers based on the multivariate regression model to predict the efficacy (DCR) of the mFOLFIRINOX regimen in patients with mPC. Statistical significance was set at *P <*0.05 (two-sided).

## Results

### Characteristics of the patients

All the 36 patients included had stage IV PC. Six patients were excluded from data analysis: four received two or fewer cycles because of intolerable toxicity, one was lost to follow-up, and one received only one cycle for other reasons. Therefore, 30 patients were included in the evaluation of ORR and DCR.

As shown in **Table 2**, the median age was 63 years. Twenty-one (70.0%) patients were male, and nine (30.0%) were female. Most patients (70.0%) were ECOG 1, and one patient was ECOG 2. Eight (26.6%) patients had tumors located in the head of the pancreas, eleven (36.7%) in the body, and eleven (36.7%) in the tail. The most common metastatic sites were lymph nodes (56.7%) and the liver (56.7%). Patients received a median of 6 cycles, and 5 patients received at least 10 cycles. No patients had CR, 8 (26.7%) patients had PR, 18 (60.0%) had SD, and 4 (13.3%) had PD. The ORR was 26.7%, and the DCR was 86.7%. The median PFS was 189 (95% CI: 136–241) days (**Supplementary Figure 1**).

**Table 2:** Baseline characteristics of the patients.

Characteristics	Pancreatic cancer cases (*n* = 30)
Age, median (range), year	63 (59-68)
Sex, *n* (%)	
Male	21 (70.0)
Female	9 (30.0)
ECOG, *n* (%)^a^	
0	8 (26.7)
1	21 (70.0)
2	1 (3.3)
BMI, median (range), kg/m^2^	20.6 (18.3-23.0)
Primary tumor location, *n* (%)	
Head	8 (26.6)
Body	11 (36.7)
Tail	11 (36.7)
Accept operation before metastasis, *n* (%)	
No	15 (50.0)
Yes	15 (50.0)
CA199, U/mL, *n* (%)	
>40	25 (83.3)
≤40	5 (16.7)
CEA, ng/mL, *n* (%)	
>5	16 (53.3)
≤5	14 (46.7)
Measurable metastatic site, *n* (%)	
Liver	17 (56.7)
Peripancreas	5 (16.7)
Lymph node	17 (56.7)
Lung	8 (26.6)
Peritoneal	7 (23.3)
Other	8 (26.7)
Clinical response, *n* (%)^b^	
CR	0
PR	8 (26.7)
SD	18 (60.0)
PD	4 (13.3)
CR + PR	8 (26.7)
CR + PR + SD	26 (86.7)

BMI, Body mass index; CA199, carbohydrate antigen 199; CEA, carcinoembryonic antigen; CR, complete response; ECOG, Eastern Cooperative Oncology Group; PD, progressive disease; PR, partial response; SD, stable disease.

The clinicopathological characteristics are expressed as the median (25th–75th percentile), and categorical data are expressed as n (%).

^a^ECOG performance status is assessed on five grades, with higher numbers indicating greater disability; a score of 0 indicates that the patient is fully active and able to carry on all pre-disease performance without restriction; a score of 1 indicates that the patient is restricted in physically strenuous activity but ambulatory and able to carry out work of a light or sedentary nature; a score of 2 indicates that the patient is ambulatory and capable of all self-care but unable to carry out any work activities; up and about more than 50% of waking hours.

^b^Clinical response was assessed according to the Response Evaluation Criteria in Solid Tumors version 1.1 (RECIST 1.1).

### Correlation between inflammatory markers and effectiveness of mFOLFIRINOX

**Figure 2:**
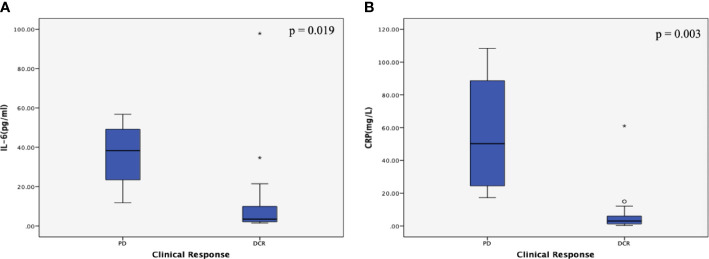
Correlations between inflammatory marker levels and the clinical response subgroup in patients with metastatic pancreatic cancer (mPC). **(A)** Correlation between interleukin (IL)-6 levels and the clinical response subgroup in patients with mPC. The serum IL-6 levels were higher in the PD group than in the DCR group (P = 0.019). **(B)** Correlation between C-reactive protein (CRP) levels and the clinical response subgroup in patients with mPC. The serum CRP levels were higher in the PD group than in the DCR group (P = 0.003). The *P* values were calculated using the Student’s *t* test. The symbol * means abnormal values in the boxplot.

Among the inflammatory markers, IL-6 and CRP were significantly and positively associated with disease progression (*r* = 0.515, *P* = 0.004; and *r* = 0.711, *P* = <0.001, respectively) (**Table 3**). As shown in **Figure 2**, the serum IL-6 and CRP levels were higher in the PD group than in the DCR group (*P* = 0.019 and *P* = 0.003, respectively). Serum albumin levels were not different between the DCR and PD groups, and the serum levels of the other inflammatory markers were similar in the two groups (**Table 3** and **Supplementary Figure 2**). Tumor markers, including CA199 and CEA, were not associated with the disease outcomes in this study (**Table 3** and **Supplementary Figure 2**).

**Table 3:** Correlation between clinicopathological markers and clinical response in patients with mPC treated with mFOLFIRINOX.

	Clinical response			*P* ^a^	Spearman correlation	*P* ^b^
	PD	DCR				
		PR	SD			
IL-6 (pg/L)				**0.012**	**0.515**	**0.004**
High (>7)	4	2	5			
Low (≤7)	0	7	12			
CRP (mg/L)				**0.001**	**0.711**	**<0.001**
High (>10)	4	1	2			
Low (≤10)	0	8	15			
Albumin (g/L)				0.611	-0.109	0.568
High (≥35)	2	6	11			
Low (<35)	2	3	6			
Neutrophils (×10^9^/L)				0.360	0.135	0.478
High (>7)	1	1	2			
Low (≤7)	3	8	15			
Lymphocytes (×10^9^/L)				1.000	-0.154	0.417
High (≥0.8)	4	8	14			
Low (<0.8)	0	1	3			
Platelets (×10^9^/L)				1.000	-0.131	0.491
High (≤303)	0	0	3			
Low (>303)	4	9	14			
CA199 (U/mL)				0.557	0.196	0.299
High (>40)	4	6	14			
Low (≤40)	0	3	3			
CEA (ng/mL)				0.550	0.237	0.208
High (>5)	4	6	12			
Low (≤5)	0	3	5			
Liver metastasis				0.613	0.145	0.444
Yes	3	4	10			
no	1	5	7			
Lung metastasis				0.550	-0.237	0.208
Yes	0	3	5			
no	4	6	12			

CA199, Carbohydrate antigen 199; CEA, carcinoembryonic antigen; CR, complete response; CRP, C-reactive protein; DCR, CR + PR + SD; IL-6, interleukin-6; PD, progressive disease; PR, partial response; SD, stable disease.

a Fisher exact test. Statistical significance was set at P <0.05 (two-sided).

b Spearman’s rank correlation coefficient. Statistical significance was set at P <0.05 (two-sided).

The bold values mean statistically significant.

### Correlation between dynamic changes in inflammation markers and clinical response to mFOLFIRINOX

**Figure 3:**
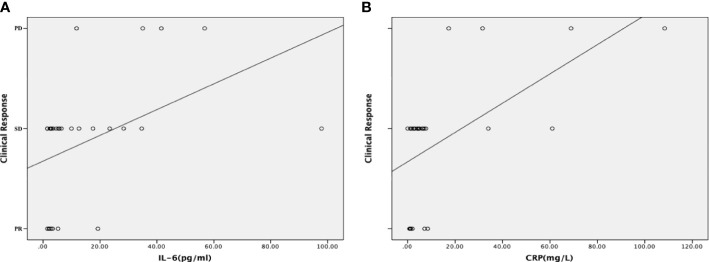
Correlations between the dynamic changes in inflammatory markers and the clinical response. **(A)** Correlation between the dynamic changes in the serum interleukin (IL)-6 level and the clinical response. The dynamic increases in the levels of IL-6 positively correlated with progressive disease (PD) during chemotherapy. **(B)** Correlation between the dynamic changes in the serum C-reactive protein (CRP) level and the clinical response. The dynamic increases in the levels of CRP positively correlated with progressive disease (PD) during chemotherapy. A scatter plot and a fitted curved were used to show the correlation more intuitively.

To further describe the correlation between levels of IL-6 and CRP and clinical response, the dynamic changes in IL-6 and CRP were explored. Dynamic increases in IL-6 and CRP significantly correlated with the clinical response (*P* = 0.005 for IL-6; *P* = 0.001 for CRP) (**Supplementary Table 1**). As shown in **Figure 3**, the dynamic changes in IL-6 and CRP levels positively correlated with PD during chemotherapy (*r*= -0.599, *P* = <0.001 for IL-6; *r* = -0.711, *P* = <0.001 for CRP).

### Correlation between IL-6 and CRP levels and the incidence of lung/liver metastasis at baseline

IL-6 was significantly and positively associated with CRP (*r* = 9.459, *P* = 0.004; and *r* = 0.562, *P* = 0.001, respectively) (**Table 4**). Since CRP is produced by the liver as part of the acute phase response, its levels correlate with cancer progression. The correlation between levels of IL-6 and CRP and the incidence of liver metastasis at baseline, as well as the correlation between the incidence of liver metastasis and the effectiveness of mFOLFIRINOX, were also explored. The correlation between levels of IL-6 and CRP and the incidence of lung metastasis was explored at the same time. The incidence of lung/liver metastasis at baseline was neither associated with baseline IL-6 or CRP levels nor with the best response to chemotherapy in this study (**Table 3** and **Supplementary Table 2**).

**Table 4:** Correlation between IL-6 and CRP levels in patients with mPC treated with mFOLFIRINOX.

	CRP (mg/L)		*P* ^a^	Correlation coefficient	*P* ^b^
	High (>10)	Low (≤10)			
IL-6 (pg/L)			0.004	0.562	0.001
High (>7)	6	5			
Low (≤7)	1	18			

CRP, C-reactive protein; IL-6, interleukin-6.

a The P values were calculated using Fisher’s exact test.

b The P values were calculated using Spearman’s rank correlation coefficient.

Statistical significance was set at P <0.05 (two-sided).

### Risk factors associated with the outcomes of patients with mPC

**Figure 4:**
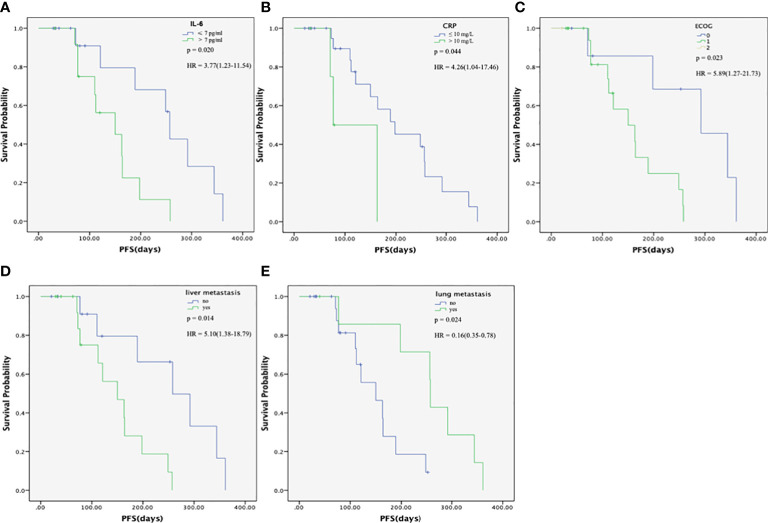
Progression-free survival (PFS) of patients with metastatic pancreatic cancer (mPC) treated with the modified FOLFIRINOX. **(A)** PFS in the interleukin (IL)-6-high and –low groups. The median PFS in the IL-6-low group was 257 (95% CI: 237-276) days, which was significantly higher than that in the IL-6-high group (median PFS, 150 days; 95% CI:47-252, P=0.020). **(B)** PFS in the C-reactive protein (CRP)-high and -low groups. The median PFS in the CRP-low group was 198 (95% CI: 42-353) days, which was significantly higher than that in the CRP-high group (median PFS, 163 days; 95% CI:28-297, P = 0.044). **(C)** PFS in the different ECOG groups. The median PFS was 292 (95% CI: 151-432) days and 150 (95% CI:81-218) days for ECOG score 0 and 1 group, respectively. **(D)** PFS according to the presence of liver metastasis. The median PFS in the non-liver metastasis group was 258 (95% CI:143-372) days, which was significantly higher than that in the liver metastasis group (median PFS, 150 days; 95% CI: 84-215, P = 0.014). **(E)** PFS, according to the presence of lung metastasis. The median PFS in the lung metastasis group was 258 (95% CI:255-260) days, which was significantly higher than that in the non-lung metastasis group (median PFS, 150 days; 95% CI: 86-213 P = 0.024). The PFS was calculated using the Kaplan-Meier method.

The univariate analyses showed that the liver metastasis (HR = 5.10, 95% CI: 1.38-18.79, *P* = 0.014), high IL-6 levels (HR = 3.77, 95% CI: 1.23-11.54, *P* = 0.020), and high CRP levels (HR = 4.26, 95% CI: 1.04-17.46, *P* = 0.044) were associated with a poorer PFS, while lung metastasis was associated with a better PFS (HR = 0.16, 95% CI: 0.35-0.78, *P* = 0.024) (**Table 5** and **Figures 4A**–**E**). The multivariate analysis revealed lung metastasis as a good independent prognostic factor (HR = 0.13, 95% CI: 0.24-0.70, *P* = 0.018) and high IL-6 levels as a poor independent prognostic factor for PFS (HR = 4.66, 95% CI: 1.32-16.37, *P* = 0.016). (**Table 3**). As shown in **Figure 4A**, the median PFS in the IL-6-low group was 257 (95% CI: 237-276) days, which was significantly higher than that in the IL-6-high group (median PFS, 150 days; 95% CI:47-252, *P* = 0.020). Tumor markers, including CEA and CA199, were not associated with PFS in this study (*P* = 0.529 and *P* = 0.146, respectively) (**Supplementary Figures 4A**, **B**). So far, the median OS of these patients had not been reached.

**Table 5:** Univariate and multivariate analyses of clinicopathological data and inflammatory markers in PFS.

Characteristics	Univariate analysis		Multivariate analysis	
	HR (95% CI)	*P* ^c^	HR (95% CI)	*P* ^d^
Age, year^a^		0.250		
>63	1 (ref)			
≤63	1.86 (0.64-5.43)			
Sex		0.624		
Female	1 (ref)			
Male	1.35 (0.40-4.48)			
ECOG^b^		**0.023**		
0	1 (ref)			
1–2	5.89 (1.27-21.73)			
BMI^a^		0.259		
>20.6	1 (ref)			
≤20.6	1.77 (0.65-4.81)			
Primary tumor location		0.735		
Head	1 (ref)			
Body	1.52 (0.41-5.56)			
Tail	1.54 (0.48-4.91)			
Operation before metastasis		0.405		
Yes	1 (ref)			
No	1.55 (0.55-4.37)			
CA199 (U/mL)		0.488		
≤40	1 (ref)			
>40	1.71 (0.37-7.81)			
CEA (ng/mL)		0.939		
≤5	1 (ref)			
>5	1.04 (0.38-2.84)			
Metastatic time		0.239		
Metachronous	1 (ref)			
Synchronous	1.92 (0.64-5.68)			
Measurable metastatic site				
Liver		**0.014**		0.132
No	1 (ref)		1 (ref)	
Yes	5.10 (1.38-18.79)		2.99 (0.72-12.44)	
Peripancreas		0.283		
No	1 (ref)			
Yes	2.27 (0.50-10.13)			
Lymph node		0.914		
No	1 (ref)			
Yes	0.94 (0.31-2.82)			
Lung		**0.024**		**0.018**
No	1 (ref)		1 (ref)	
Yes	0.16 (0.35-0.78)		0.13 (0.24-0.70)	
Peritoneal		0.982		
No	1 (ref)			
Yes	1.01 (0.27-3.71)			
Other		0.984		
No	1 (ref)			
Yes	1.01 (0.32-3.14)			
IL-6 (pg/mL)		**0.020**		**0.016**
≤7	1 (ref)		1 (ref)	
>7	3.77 (1.23-11.54)		4.66 (1.32-16.37)	
CRP (mg/L)		**0.044**		
≤10	1 (ref)			
>10	4.26 (1.04-17.46)			
Neutrophils (×10^9^/L)		0.114		
≤7	1 (ref)			
>7	4.01 (0.71-22.52)			
Lymphocytes (×10^9^/L)		0.829		
≥0.8	1 (ref)			
<0.8	1.25 (0.16-9.67)			
Platelets (×10^9^/L)		0.533		
≤303	1 (ref)			
>303	1.65 (0.35-7.46)			
Albumin (g/L)		0.061		
≥35	1 (ref)			
<35	3.16 (0.94-10.58)			

BMI, Body mass index; CA199, carbohydrate antigen 199; CEA, carcinoembryonic antigen; CI, confidence interval; CRP, C-reactive protein; ECOG, Eastern Cooperative Oncology Group; HR, hazard ratio; IL-6, interleukin-6.

^a^Using the median value as a cutoff value.

^b^ECOG performance status is assessed on five grades, with higher numbers indicating greater disability; a score of 0 indicates that the patient is fully active and able to carry on all pre-disease performance without restriction; a score of 1 indicates that the patient is restricted in physically strenuous activity but ambulatory and able to carry out work of a light or sedentary nature; a score of 2 indicates that the patient is ambulatory and capable of all self-care but unable to carry out any work activities; up and about more than 50% of waking hours.

^c^Univariable analyses were performed using the Kaplan–Meier method.

^d^Multivariable analysis was performed using a stepwise forward Cox regression (likelihood ratio, enter P <0.05, remove P >0.10) with significant markers from the univariate analysis (P < 0.05). The bold values mean statistically significant.

### ROC curve of the predictive model for the efficacy of mFOLFIRINOX in mPC

**Figure 5:**
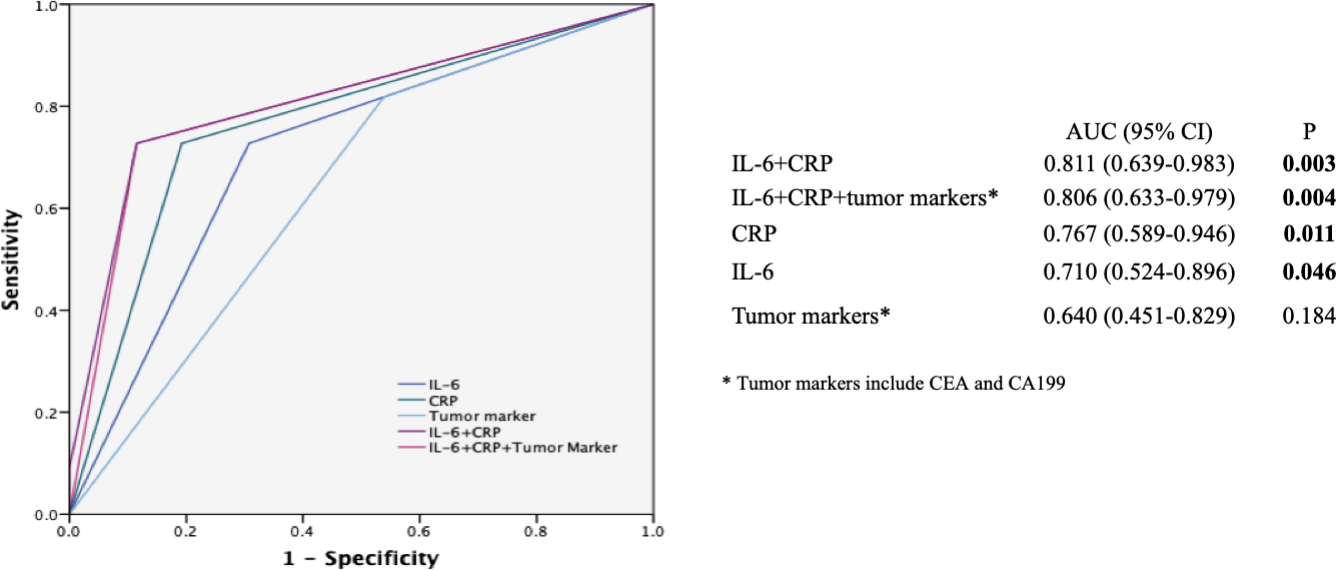
Receiver operating characteristic (ROC) curves for predicting the efficacy of the mFOLFIRINOX regimen. ROC analyses of the prediction of the efficacy of mFOLFIRINOX using the inflammatory marker model, the tumor marker model, and the combined inflammatory and tumor marker model. The ROC analysis showed that the combination of IL-6 and CRP (AUC: 0.811, 95% CI: 0.639-0.983, P = 0.003) had a higher AUC compared with CRP alone (AUC: 0.767, 95% CI: 0.589-0.946, P = 0.011), IL-6 alone (AUC: 0.710, 95% CI: 0.524-0.896, P = 0.046), tumor markers (AUC: 0.640, 95% CI: 0.451-0.829. P = 0.184) alone, and their combination (IL-6 + CRP + tumor markers, AUC: 0. 806, 95% CI: 0.633-0.979, P = 0.004). AUC, Area under the curve. The symbol * is the annotation of tumor markers.

The inflammatory markers (IL-6 and CRP) were used for constructing a model to predict the effectiveness (DCR or not) of the mFOLFIRINOX regimen in patients with mPC. Concerning the possible predictive value of tumor biomarkers for the treatment response indicated in previous studies (8, 9, 24), CEA and CA199 were also added. As shown in **Figure 5**, the ROC analysis showed that the combination of IL-6 and CRP (AUC: 0.811, 95% CI: 0.639–0.983, *P* = 0.003) had a higher AUC compared with CRP alone (AUC: 0.767, 95% CI: 0.589–0.946, *P* = 0.011), IL-6 alone (AUC: 0.710, 95% CI: 0.524–0.896, *P* = 0.046), tumor markers (AUC: 0.640, 95% CI: 0.451–0.829, *P* = 0.184) alone, and their combination (IL-6 + CRP + tumor markers, AUC: 0.806, 95% CI: 0.633–0.979, *P* = 0.004).

## Discussion

In this single-institution retrospective analysis, the median PFS was 189 days and the DCR was 86.7% among 30 patients with mPC treated with mFOLFIRINOX, which were comparable with previously reported findings (4–7). High levels of IL-6 and CRP were associated with poor treatment response. The dynamic increase in IL-6 and CRP levels during treatment was associated with a poor progression of mPC. High IL-6 was a predictor of poor PFS, while lung metastasis was a predictor of better PFS in the multivariate analysis. The model combining IL-6 with CRP showed a better value in predicting the outcomes of patients with mPC treated with mFOLFIRINOX. Furthermore, this study was novel in reporting the associations between the dynamic changes in serum IL-6 and CRP levels and clinical response.

CRP is synthesized by the hepatocytes and is one of the most commonly used markers to reflect systemic inflammation. In this study, the serum CRP levels were associated with the tumor response, which was consistent with previous findings. Indeed, Nurmi et al. (25) described the combination of CRP and CA19-9 as a useful prognostic marker in evaluating disease-specific survival of surgically treated patients with PC. Liu et al. (12) revealed an elevated CRP/albumin ratio as an independent factor for poor prognosis with the cutoff value of 0.180 in PC patients. The prognostic significance of the CRP/albumin ratio existed in Stages III and IV PC patients and had no connection with the primary tumor location. Mitsunaga et al. (16) investigated the serum levels of CRP and clinical outcomes in patients with APC treated with first-line gemcitabine monotherapy; low, intermediate, and high CRP levels were associated with a good, intermediate, and poor response to chemotherapy. Besides, the elevated CRP level was found to be statistically significant in patients with mPC receiving first-line chemotherapy, including FOLFIRINOX, monotherapy with gemcitabine, and doublet chemotherapy with gemcitabine in combination with cisplatin or oxaliplatin; it was also significantly associated with shorter PFS and OS (26, 27). These results implied that an underlying inflammatory state might play a role in resistance to systemic therapy or in increased tumor aggressiveness, thus supporting the conclusion of the present study that CRP was a possible biomarker predicting the efficacy of the mFOLFIRINOX regimen. This should be further explored in future studies.

IL-6 regulates the secretion of vascular endothelial growth factor (VEGF) in PC cells, thereby stimulating angiogenesis and tumor vascularization, resulting in lymphatic and distant metastasis and disease progression. High IL-6 levels predict poor PFS and poor response to gemcitabine monotherapy in patients with APC (15). A phase II trial reported that higher serum IL-6 levels before treatment were associated with a reduced response to therapy and worse OS in patients with APC treated with gemcitabine and curcumin (28). This study suggested that the serum levels of IL-6 were closely related to poor response and prognosis of mPC treated with mFOLFIRINOX, which was supported by the aforementioned studies. In consideration of the complexity of the low half-life of IL-6, it may not be an ideal biomarker in clinical practice. A high serum level of CRP was reported to be related to a high serum IL-6 level in patients with treatment-naive APC (29). CRP may be a better substitute marker for IL-6 in predicting the efficacy of the mFOLFIRINOX regimen and PFS in patients with mPC. In the present study, IL-6 was significantly and positively associated with CRP in mPC patients who received the mFOLFIRINOX regimen as first-line chemotherapy. High levels of IL-6 and CRP were associated with poor treatment response, and they were also proved to be a predictor in the univariate analyses. The AUC of CRP alone was also higher than that of IL-6 alone. However, CRP was not associated with PFS in the multivariate analysis. The small sample size might have played a role in this result. The relationship between CRP and IL-6 in predicting PFS in patients with mPC should be further explored in future studies.

IL-6 induces metastasis through programming hematopoietic stem and progenitor cells toward tumor-supporting metastatic cells (30). Additionally, IL-6 affects monocyte-dendritic progenitors to differentiate into metastasis-promoting cells, thereby promoting tumor aggressiveness (30). An elevated CRP level may reflect a non-specific inflammatory response to tumor necrosis, which in turn indicates the levels of inflammatory cytokines such as IL-6. The production of inflammatory cytokines leads to the promotion of adhesion of circulating tumor cells to the vascular endothelium of distant organs by enhancing the E-selectin expression. This results in a microenvironment that favors tumor metastases (31, 32).The most common sites of metastasis in mPC are the liver, lung, regional lymph nodes, and peritoneum. Different metastatic patterns may involve different tumor biology and prognosis (33).

Kurokawa et al. (32) showed that liver metastasis occurred more frequently in high serum levels of CRP group in patients with pT2-T4 gastric cancer who underwent R0 resection. Kim et al. (34)revealed that IL-6 was significantly direct correlated with CRP, and it was an independent risk factor for progression to extensive hepatic metastasis in patients with PC. The correlation between lung metastasis and IL-6/CRP levels has not been found yet in published studies. In our study, IL-6 was significantly and positively associated with CRP in mPC patients, which was in line with Kim et al’s study. However, the incidence of lung/liver metastasis at baseline was neither associated with baseline IL-6 or CRP levels nor with the best response to chemotherapy. The small sample size might have played a role in this result. The relationship between the lung/liver metastasis and CRP/IL-6 levels in predicting chemotherapy efficacy in mPC patients should be further explored in future studies.

Liver metastasis was a poor prognostic factor in this study, as supported by the MPACT study (35), while the presence of lung metastasis was considered as a good prognostic factor, which was also observed in other studies, but the mechanisms remain unclear.

Lung metastasis as the primary recurrence was reported as a favorable prognostic factor in patients with relapsed PC (36). Isolated lung metastases were demonstrated as a better prognosticator for OS in stage IV PC treated with palliative chemotherapy (37). Kruger et al. showed that limited disease (defined as metastatic disease of fewer than 10 metastases) confined to one lung might predict favorable outcomes in patients with PC without metastases to other organs (38). In addition, Lovecek et al. examined patients with PC who developed metachronous pulmonary metastases as the first site of recurrence after the curative-intent surgery. His result showed that patients with pulmonary metastases, including isolated pulmonary oligometastases, isolated pulmonary multiple metastases, and pulmonary metastases accompanied by other metastases, had prior DFS and OS compared with patients with nonpulmonary metastases (39). Patients with lung metastasis from PC had a better prognosis compared with those with other site metastases, which is still a controversial issue. Lung metastasis might confer less clinical-related complications compared with the complications that might be caused by local recurrence or liver metastasis, including biliary obstruction or gastric outlet obstruction. It might explain the better PFS or OS in patients with lung metastases. Patients with lung metastasis as the primary recurrence usually had lower pT category and less vascular invasion compared with patients with other metastases, and it might influence the prognosis (36). In the present study, all of the patients with lung metastasis also had other metastases. Patients with lung metastasis accompanied by other metastases had longer PFS compared with patients with non-lung metastasis. The difference between isolated lung metastasis and lung metastasis accompanied by other metastases in PFS of patients with mPC should be further studied in the future.

Circulating neutrophils (innate immune system) are thought to play a tumor-promoting role, while lymphocytes (adaptive immune system) play an anti-tumor role. High neutrophil counts were associated with a worse prognosis in patients with various cancers (40, 41). Angiogenesis is one of the most important causes of tumor proliferation and metastasis. Platelets contribute to tumor vascular growth through a number of platelet-derived angiogenic factors such as vascular endothelial growth factor (VEGF), platelet-derived growth factor, and hepatocyte growth factor. High platelet counts were associated with a worse prognosis in PC (42) and other cancer types (23, 43). Serum albumin is an important indicator of nutritional status. Hypoalbuminemia usually occurs in combination with poor performance status, weight loss, and nutritional deficiency, which negatively affect the prognosis of cancer patients. The relationship between hypoalbuminemia and poor survival may also depend on the systemic inflammatory response (44). A low serum albumin level was reported as a significant independent predictive factor for PFS in cancer patients (45–47). CEA and CA199 have been demonstrated to be predictive markers for the response to chemotherapy in PC (8, 48), with elevated serum values leading to poor OS (49, 50). Several studies showed that CA199 reduction during chemotherapy was a potential predictor of efficacy (51, 52). However, in this study, neutrophils, lymphocytes, platelets, albumin, CEA, and CA199 did not correlate with the clinical response to mFOLFIRINOX or with PFS. In this study, the cutoff values were determined using the normal ranges, while previous studies used statistical methods to determine the cutoff. The sample size, as well as the type of therapy, might also play a role.

In the present study, the results suggested that IL-6 and CRP could effectively predict the clinical response to mFOLFIRINOX in mPC. CEA and CA199 were added to the predictive model because they are known to be markers of treatment response in PC (51, 52), but they did not improve the predictive value of IL-6 and CRP; the combination of IL-6 and CRP showed the best predictive value. This was supported by the fact that high IL-6 and CRP levels were associated with poorer PFS and that increases in IL-6 and CRP levels during treatment were associated with poorer prognosis. Nevertheless, models remain to be determined and improved for the prognosis of mPC. For now, the results suggested that patients with low IL-6 and low CRP levels before treatment might be those who would respond to mFOLFIRINOX. Additional studies are needed to refine these results.

This study had some limitations. The small sample size of this study might have prevented achieving sufficient power to detect survival differences between subgroups, which might be regarded as a major limitation. Additional data from other databases should be validated as well to provide a more powerful conclusion to avoid bias. However, no comparable published studies support a meta-analysis for external effectiveness in chemotherapy-naive mPC patients treated with the mFOLFIRINOX regimen as first-line chemotherapy. The results need to be validated using more samples in prospective studies. In addition, the retrospective nature of the study limited the analyzable data to those contained in the charts. A prospective cohort study may answer many questions regarding the markers of good response to mFOLFIRINOX.

In conclusion, higher IL-6 or CRP levels before or during chemotherapy positively correlated with PD. Higher serum levels of IL-6 and CRP might be predictors of a poor response to mFOLFIRINOX chemotherapy in patients with mPC. The combination of IL-6 and CRP might predict the response to mFOLFIRINOX. The relationship of inflammatory markers with the therapeutic effect of mFOLFIRINOX in patients with mPC deserves to be further investigated in the future.

## Data availability statement

The raw data supporting the conclusions of this article will be made available by the authors, without undue reservation.

## Ethics statement

Ethical review and approval was not required for the study on human participants in accordance with the local legislation and institutional requirements. Written informed consent for participation was not required for this study in accordance with the national legislation and the institutional requirements.

## Author contributions

(I) Conception and design, FZ; (II) Administrative support, none; (III) Provision of study materials or patients, FZ and QL; (IV) Collection and assembly of data, FS, WZ, CL, SH, FW, and JW; (V) Data analysis and interpretation, FS; (VI) Manuscript writing, all authors; (VII) Final approval of manuscript, all authors.

## Conflict of interest

The authors declare that the research was conducted in the absence of any commercial or financial relationships that could be construed as a potential conflict of interest.

## Publisher’s note

All claims expressed in this article are solely those of the authors and do not necessarily represent those of their affiliated organizations, or those of the publisher, the editors and the reviewers. Any product that may be evaluated in this article, or claim that may be made by its manufacturer, is not guaranteed or endorsed by the publisher.
