# Supplementary material for: Serum levels of IL-6 and CRP can predict the efficacy of mFOLFIRINOX in patients with advanced pancreatic cancer
Source: Front Oncol. 2022 Jul 29;12:964115. doi: 10.3389/fonc.2022.964115 (PMC9372918; doi:10.3389/fonc.2022.964115)
Supplement: Supplementary file 1 [file DataSheet_1.docx]

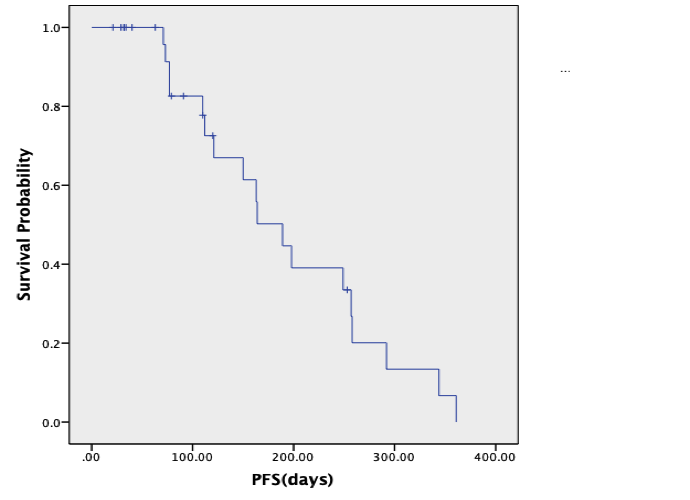


**Supplementary Figure 1. Progression-free survival (PFS) of all patients with metastatic pancreatic cancer who received the modified FOLFIRINOX regimen.** The median PFS was 189 days in the evaluable patients (95% confidence interval: 136-241).

| **a**  **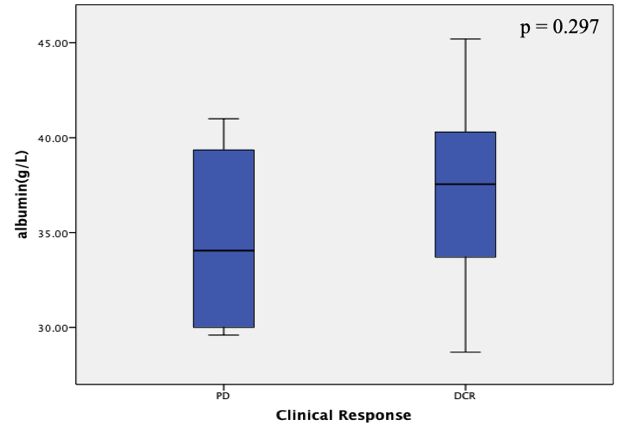**  **c**  **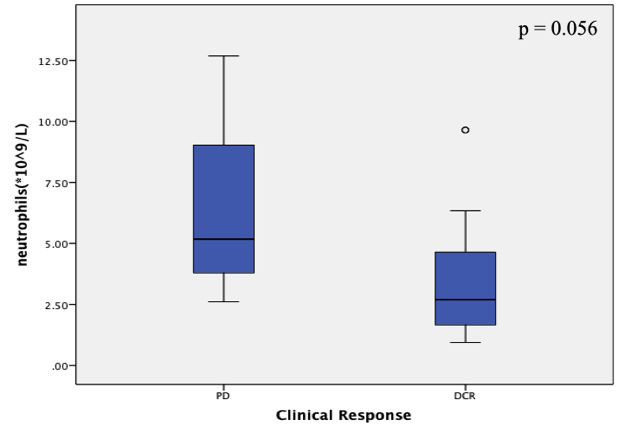** | **b**  **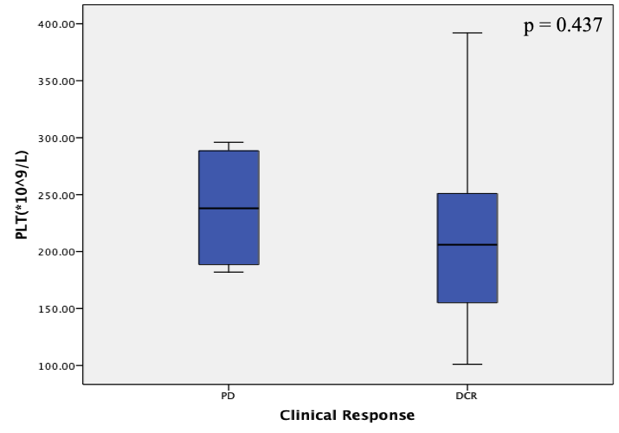**  **d**  **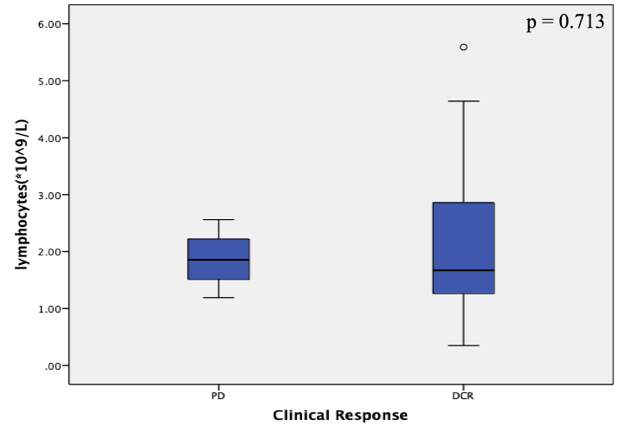** |
| --- | --- |
| **Supplementary Figure 2. Correlation between inflammatory marker levels and different clinical responses in patients with metastatic pancreatic cancer (mPC).** (a) Correlation between albumin levels and the clinical response subgroup in patients with mPC. Serum albumin levels were not different between the DCR and PD groups. (b) Correlation between PLT levels and the clinical response subgroup in patients with mPC. The serum PLT levels were similar in the two groups. (c) Correlation between neutrophils levels and the clinical response subgroup in patients with mPC. The neutrophils levels were similar in the two groups. (d) Correlation between lymphocyte levels and the clinical response subgroup in patients with mPC. The lymphocyte levels were similar in the two groups. The *P* values were calculated using the Student *t* test. | |

| **a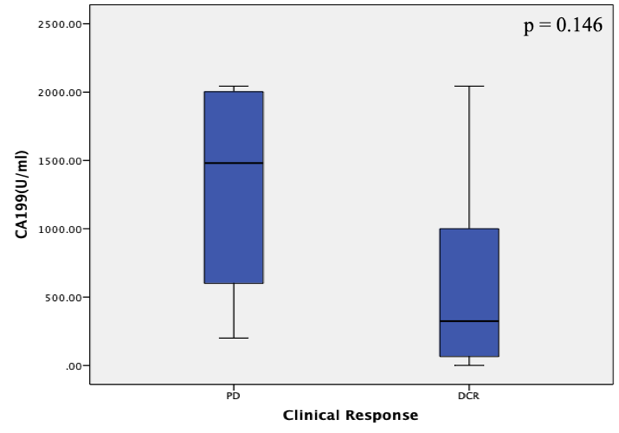** | **b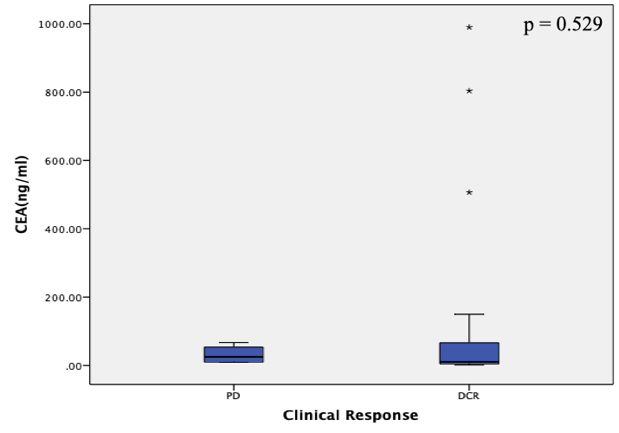** |
| --- | --- |
| **Supplementary Figure 3.** **Correlation between serum tumor marker levels and** **different clinical responses in patients with metastatic pancreatic cancer (mPC).** (a) Correlation between CA199 levels and the clinical response subgroup in patients with mPC. (b) Correlation between CEA levels and the clinical response subgroup in patients with mPC. Tumor markers, including CA199 and CEA, were not associated with the disease outcomes in this study. The *P* values were calculated using the Student *t* test. | |

| **a 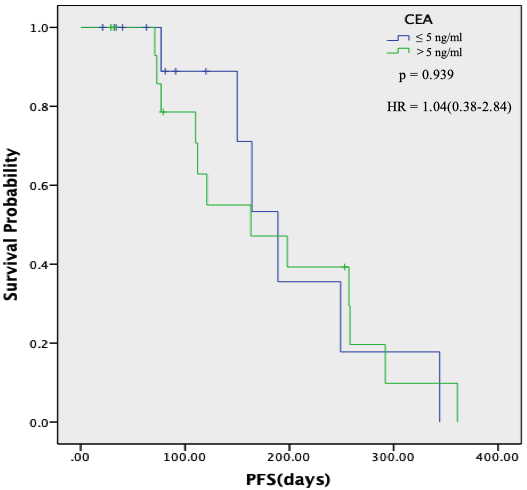** | **b 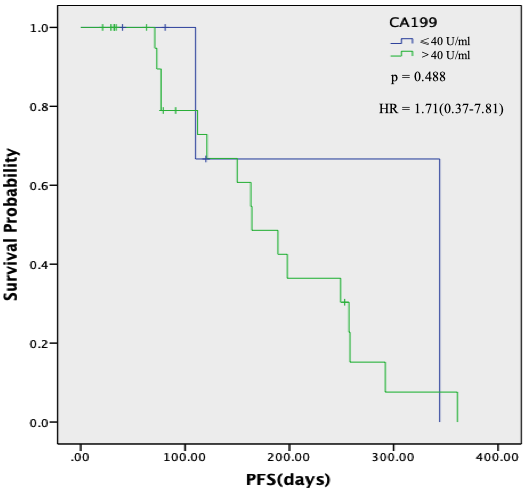** |
| --- | --- |
| **Supplementary Figure 4. Progression-free survival (PFS) of patients with metastatic pancreatic cancer (mPC) and treated with the modified FOLFIRINOX regimen in different groups.** (a) PFS in the CEA-high and CEA-low groups. The median PFS in the CEA-high group was 163 (95% CI:30-295) days, while in the CEA-low group was 189 (95% CI:146-231) days. (b) PFS in the CA199-high and CA199-low groups. The median PFS in the CA199-high group was 164 (95% CI:113-214) days, while in the CA199-low group was 344 days. The PFS was calculated using the Kaplan–Meier method. | |

| **Supplementary Table 1. Correlation between dynamically changing inflammatory markers and clinical response in patients with mPC treated with mFOLFIRINOX** | | | | | | |
| --- | --- | --- | --- | --- | --- | --- |
| Dynamic increase | Clinical response | | | *P*^a^ | Correlation coefficient | *P*^b^ |
|  | PD | DCR | |  |  |  |
|  |  | PR | SD |  |  |  |
| IL-6 |  |  |  | 0.005 | –0.599 | <0.001 |
| Increase^*^ | 4 | 0 | 5 |  |  |  |
| No increase | 0 | 8 | 13 |  |  |  |
| CRP |  |  |  | 0.001 | –0.711 | <0.001 |
| Increase^*^ | 4 | 0 | 3 |  |  |  |
| No increase | 0 | 8 | 15 |  |  |  |
| CR, Complete response; CRP, C-reactive protein; DCR, CR + PR + SD; IL-6, interleukin-6; PD, progressive disease; PR, partial response; SD, stable disease.  ^*^Increase was defined as two or more consecutive increases in the levels of tested markers compared with the last testing without a cutoff threshold.  ^a^The *P* values were calculated using Fisher’s exact test.  ^b^ The *P* values were calculated using Spearman’s rank correlation coefficient.  Statistical signiﬁcance was set at *P* <0.05 (two-sided). | | | | | | |

| **Supplementary Table 2.** Correlation between inflammatory-related markers and incidence of lung/liver metastasis in mPC patients treated with mFOLFIRINOX | | | | | | | | | | |
| --- | --- | --- | --- | --- | --- | --- | --- | --- | --- | --- |
|  | Liver metastasis | | *P*^a^ | Spearman correlation | *P*^b^ | Lung metastasis | | *P*^a^ | Spearman correlation | *P*^b^ |
|  | Yes | No |  |  |  | Yes | No |  |  |  |
| IL-6 (pg/L) |  |  | 0.708 | 0.107 | 0.574 |  |  | 0.672 | -0.146 | 0.441 |
| High (>7) | 7 | 4 |  |  |  | 2 | 9 |  |  |  |
| Low (≤7) | 10 | 9 |  |  |  | 6 | 13 |  |  |  |
| CRP (mg/L) |  |  | 0.104 | 0.323 | 0.081 |  |  | 0.143 | -0.333 | 0.072 |
| High (>10) | 6 | 1 |  |  |  | 0 | 7 |  |  |  |
| Low (≤10) | 11 | 12 |  |  |  | 8 | 15 |  |  |  |
| CRP, C-reactive protein; IL-6, interleukin-6;  ^a^Fisher exact test. Statistical signiﬁcance was set at *P* <0.05 (two-sided).  ^b^Spearman’s rank correlation coefficient. Statistical signiﬁcance was set at *P* <0.05 (two-sided). | | | | | | | | | | |

| **Supplementary Table 3. Variables in the multivariate analysis** | | | | | | | | | |
| --- | --- | --- | --- | --- | --- | --- | --- | --- | --- |
|  | | B | SE | Wald | df | Sig. | Exp(B) | 95% CI for Exp(B) | |
|  |  |  |  |  |  |  |  | Lower | upper |
| Step 1 | Liver metastasis | 1.631 | 0.665 | 6.020 | 1 | 0.014 | 5.108 | 1.388 | 18.795 |
| Step 2 | Lung metastasis | -1.486 | 0.807 | 3.390 | 1 | 0.066 | 0.226 | 0.047 | 1.101 |
|  | Liver metastasis | 1.370 | 0.679 | 4.065 | 1 | 0.044 | 3.935 | 1.039 | 14.905 |
| Step 3 | Lung metastasis | -1.721 | 0.873 | 3.885 | 1 | 0.049 | 0.179 | 0.032 | 0.990 |
|  | Liver metastasis | 1.096 | 0.727 | 2.273 | 1 | 0.132 | 2.993 | 0.720 | 12.444 |
|  | IL6 | 1.304 | 0.666 | 3.830 | 1 | 0.050 | 3.685 | 0.998 | 13.606 |
| Step 4 | lung | -2.032 | 0.856 | 5.631 | 1 | 0.018 | 0.131 | 0.024 | 0.702 |
|  | IL6 | 1.540 | 0.641 | 5.775 | 1 | 0.016 | 4.663 | 1.328 | 16.371 |
| Statistical signiﬁcance was set at *P* <0.05. | | | | | | | | | |
